# Tribology of bio-inspired nanowrinkled films on ultrasoft substrates

**DOI:** 10.5936/csbj.201303002

**Published:** 2013-05-08

**Authors:** Juergen M. Lackner, Wolfgang Waldhauser, Lukasz Major, Christian Teichert, Paul Hartmann

**Affiliations:** a JOANNEUM RESEARCH Forschungsges.mb.H., Institute for Surface Technologies and Photonics, Funtional Surfaces, Leobner Straße 94, A-8712 Niklasdorf, Austria; b Polish Academy of Sciences, Institute for Metallurgy and Material Sciences (IMIM-PAN), ul. Reymonta 25, 30-059 Krakow, Poland; c University of Leoben, Institute for Physics, Franz-Josef-Straße 18, A-8700 Leoben, Austria

**Keywords:** Biomimetic, skin deformation, magnetron sputtering, polycarbonate, thermoplastic polyurethane, titanium nitride, diamond-like carbon

## Abstract

Biomimetic design of new materials uses nature as antetype, learning from billions of years of evolution. This work emphasizes the mechanical and tribological properties of skin, combining both hardness and wear resistance of its surface (the stratum corneum) with high elasticity of the bulk (epidermis, dermis, hypodermis). The key for combination of such opposite properties is wrinkling, being consequence of intrinsic stresses in the bulk (soft tissue): Tribological contact to counterparts below the stress threshold for tissue trauma occurs on the thick hard stratum corneum layer pads, while tensile loads smooth out wrinkles in between these pads. Similar mechanism offers high tribological resistance to hard films on soft, flexible polymers, which is shown for diamond-like carbon (DLC) and titanium nitride thin films on ultrasoft polyurethane and harder polycarbonate substrates. The choice of these two compared substrate materials will show that ultra-soft substrate materials are decisive for the distinct tribological material. Hierarchical wrinkled structures of films on these substrates are due to high intrinsic compressive stress, which evolves during high energetic film growth. Incremental relaxation of these stresses occurs by compound deformation of film and elastic substrate surface, appearing in hierarchical nano-wrinkles. Nano-wrinkled topographies enable high elastic deformability of thin hard films, while overstressing results in zigzag film fracture along larger hierarchical wrinkle structures. Tribologically, these fracture mechanisms are highly important for ploughing and sliding of sharp and flat counterparts on hard-coated ultra-soft substrates like polyurethane. Concentration of polyurethane deformation under the applied normal loads occurs below these zigzag cracks. Unloading closes these cracks again. Even cyclic testing do not lead to film delamination and retain low friction behavior, if the adhesion to the substrate is high and the initial friction coefficient of the film against the sliding counterpart low, e.g. found for DLC.

## 1. Introduction

Skin is the heaviest organ of all animals (e.g. human: ∼16% of body weight) being designed by nature as a three-layer, semi-dense barrier of the organism to the surrounding. It bridges brilliantly the demands of flexibility (adaptability to the underlying surface) and hardness (tribological resistance) by wrinkled and partly fractured surfaces. Additionally, high local pressure sensitivity is enabled by such a grooved structure, found for less regularly structure for human skin but for highly ordered structures for a wide variety of animal skins (e.g. on tree fog toe pads by hexagonal arrays of epithelial cells) [1–3]. Human skin is composed of a hard layer (stratum corneum) on a soft, compliant substrate (epidermis with lucidum, granulosum, spinosum, germinativum, papillary and reticular dermis and hypodermis): The 10 to 25 µm thick stratum corneum layer with an elastic modulus between 50 and 400 MPa (depending on indentation depth) is built of 10 to 20 layers of non-viable, keratinized corneocyte cells [4–7]. Mechanically, stratum corneum is described of corneocytes being “bricks”, which are bound together by 0.1–0.3 µm thin lipid-rich “mortar” (intercellular lipids and degraded desmosomal protein junctions) [6]. The compliant skin layers below are together 1 to 4 mm thick and have an elastic moduli < 1 MPa [8]. Skin topography is widely influenced by wrinkles, which form due to permanent intrinsic tensile stresses in the substrate (reticular dermis). Three hierarchical wrinkle structures with 70 – 200 µm (primary lines, “Langer's lines”), 20 – 70 µm (secondary lines) and <10 µm depth are found, covering the whole skin and enabling simultaneously surface hardness, tribological resistance, and flexibility [9]. Generally, wrinkle depth and density is adapted to the required deformability and tribological resistance: The thicker the skin and the larger its demanded deformability, the deeper and more dense wrinkles are. While the larger wrinkles are forming lines (e.g. Langer lines), the smallest wrinkle structure separates groups of corneocytes.

Under mechanical forces, the skin surface can extend without loading the cells by reversible smoothing of wrinkles. In a stress–strain curve, this results in a toe region [10, 11]. The direction for the higher extensibility is perpendicular to the direction of the primary line wrinkles with ∼40% elongation in the toe region compared to ∼20% along the wrinkles. As a general consequence, the stratum corneum hardly experiences elongation stresses but only unfolds under typical cyclic loading *in vivo*. Further straining of skin leads to straightening and alignment of the fibrous component in dermis, being visible by a linear region in the stress–strain curve [12, 13]. Focusing on the fracture of stratum corneum, plastic deformation starts after 10% extension with irreversible elongation [14]. In dry stratum corneum, cracking occurs at the end of this phase of low slope in the stress–strain curve, while a strain hardening phase occurs in hydrated stratum corneum, which is characterized by higher fiber mobility and differences in the intercellular lipid composition. This final rupture is always extracellular and most likely at the desmosomes [15]. Wu et al. [16] found 0.7 MPa peak stress for fracture of dry stratum corneum, which decreasing at higher skin hydration. At higher strains, stresses decayed due to continuing deformation of viable epidermis and dermis. The surface morphology of the fractured skin surface reveals a separation of stratum corneum islands (compare to [16]): Channeling cracks form around these islands through the whole thickness of stratum corneum. Predominantly, these extracellular channeling cracks follow the third-order wrinkles around groups of corneocytes.

Under tensile stresses, this channeling process does not arrest until it encounters another channel or an edge, creating a connected channel network [17]. Sources of channel networks at higher stresses are surface cracks starting from a flaw even at lower stresses, based on fracture mechanics based cracking models.

Tribologically, the stack of connected layers, varying in elasticity, shear strength and continuity (e.g. by wrinkles), show at minimal lubrication conditions (without any sweat, lipids, etc.), friction coefficients of 0.6 against paper, 1.6 against polyethylene or 2.6 against polycarbonate [18]. Such high coefficients of friction are due to high skin viscoelasticity [19, 20]. Lubricated conditions and filling of grooves with liquids (water, sweat, lipids) may change adhesion by capillary forces. Higher normal forces general decrease friction coefficients [21]. Friction generally leads to shear stresses, under which the stack of skin layers (except the topmost hard stratum corneum) behaves like a viscous fluid [22]. As described above and shown for tribological contact in [21], wrinkles elastically compact under compressive loading in front of a slider and smooth out under tensile loading behind the slider. Cyclic tribological loading under such conditions can result in layer-by-layer wear of the 10 to 20 anucleated corneocyte layers in the stratum corneum, whereby layer delamination is found along the cornified proteins acting as glue between these cells. If the occurring tribological tensile strains are too high and elastic compliance of skin is exhausted, plastic deformation (tissue trauma) in the subsurface layers may occur. Tensile fracture behind the moving counterbody can results in intercellular fracture along larger wrinkles. Transcellular failure is rather implausible.

Applying such a biomimetic concept for obtaining both flexibility and hardness for engineering materials gathers increasing interest, e.g. for the tribological protection of soft materials like polymers and elastomers. Generally, these materials are distinguished candidates for low weight design in mechanical engineering, but lack on mechanical surface strength and consequently tribological resistance and generally possess high friction coefficients. Their high elasticity causes large surface deflections during compressive loading, e.g. found in sliding contacts: Hard, stiff, and sharp counterparts (like e.g. mineral grains) are deeply incising, ploughing, and scratching the polymer surface. Mechanically, material is piled up in front of the moving counterpart as well as on the sides of the residual groove for polymers with high plasticity and low strain hardening [23]. Contrary, highly elastic polymers lead to sinking-in in front of the sliding counterpart. Viscoelastic and viscoplastic effects reduce the size of this groove time-dependently after tribological loading (scratching). Due to a close correlation to polymer strain hardening, the minimal groove size is found for polymer materials being hard and elastic at the same time [23]. Nevertheless, too high plastic strain finally results in material failure – e.g. microcracking, fatigue, and detachment of wear particles [24].

Coating deposition on polymers with materials of higher tribological resistance like hard films has high potential to optimize scratch and tribological resistance, following the mentioned principle for combined hard and elastic materials. The surface hardness increases, while the viscoelastic behavior of the polymer bulk is preserved. Nevertheless, hard films have poor elasticity and struggle with high deflection of soft polymer substrates. Highest tensile stress and strain levels close to the surfaces frequently exceed elastic and plastic deformability of hard films. Films immediately fail by cracking and / or delamination, if the film thickness is insufficient thick for load support. Cohesive fracture of hard films starts from the interface to the soft material and run towards the film surface. Further cohesive film cracks form in the bulged region around the indenter contact and run in opposite direction. The extent of such cohesive film as well as for subsequent adhesive fracture at the interface to the polymer depends on the film material (mechanical properties) and the film adhesion to the substrate (type of chemical and physical bonding). High friction between the sliding counterpart and the film hasten failure by residual shear stresses [25–28].

To overcome these limitations for thin films, we started biometically inspired research based on deformation of human skin. In former works, we found, that wrinkles are main element in topography formation of thin films on polymers, if they are deposited by physical vapor deposition (PVD) or plasma-activated chemical vapor deposition (PACVD) techniques under low temperature (room temperature) and high energetic conditions (high content of ions or kinetic particles in plasma) [29–31]. The influence of high energetic conditions was intensively studied by the authors for a variety of thin films (titanium, titanium nitride (TiN), precious metals, diamond-like carbon (a-C:H), etc.) on different polymers (polycarbonate (PC), thermoplastic polyurethane (PU), polyamide, polyimide, etc.). Such conditions generally lead to high intrinsic compressive film stresses due to high densities of lattice defects (deposited contaminations, ultra-fine grain size). Briefly, wrinkles occur by relaxation of these intrinsic stresses by a common deformation of the substrate surface zone and the thin film, whereby similarities in the mechanics of wrinkle formation (intrinsic stresses) as well as in the deformation behavior were found between skin and thin films [32]. Generally, a hierarchical wrinkle structure forms due to stiffening of the surface during deposition: Higher deformation resistance for intrinsic stress relaxation leads to the introduction of additional wrinkle structures of much larger wavelength. Initially occurring wrinkles are on sub-micrometer scale (“nano-wrinkles”), later formed hierarchical overstructures on micrometer scale [31]. In comparison to human skin, these hierarchically formed wrinkles on coated polymers have about 2 to 3 orders of magnitude lower size [32]. Under low strains, wrinkles can smooth out elastically [33]. Higher strains result in fracture under tensile stresses, whereby the cracks run zigzag on the micrometer scale along wrinkle grooves (the areas of lowest strength) and follow rather perpendicular to the tensile stress direction on the millimeter scale [32]. Deformation (stretching) is focused locally in the polymer below these zigzag crack bands. Film fragments between the crack bands are rather unstressed as well as the substrate surface below these fragments. Doubling the strain leads to a higher density of crack bands, while unloading closes the cracks. Adhesion of all these inorganic films on the polymers is guaranteed by a gradient interface (pseudodiffusion interface), formed by implantation of high-energetic metal atoms (up to 100's eV ionic and/or kinetic energy) in the polymer substrate during the initial phase of deposition. These metal atoms are found in up to 150 nm depth in X-ray photoelectron spectroscopy studies (shown in [29]), binding there to oxygen atoms [34] but also (revealed by Fourier-transformed infrared spectroscopy) to atoms in polymer chains [32].

Goal of this work is to figure out the influence of wrinkling on tribology (friction and wear resistance) of these compounds, whereby we will not lose track of the biomimetic comparison. Tribological conditions of scratching (ploughing) of sharp counterparts and sliding of smooth balls under low loads (mN) are applied in these investigations, going far beyond the state-of-the-science of tribology on (ultra-)thin, wrinkled hard films on (ultra-)soft substrates: Kim et al. [35] showed wrinkling influences on tribology for ultra-thin, hydrogen containing amorphous carbon (diamond-like carbon, DLC, a-C:H) coated surfaces on soft polydimethylsiloxane polymer. Low friction was found for higher film thickness (>200 nm), while high friction and strong stick-slip effects occurred for thin films (<150 nm), which is explained by elastic deformation of the soft substrate under the normal load. Nevertheless, sliding of the steel ball on the nanostructures generally lead under the applied high loads and high sliding velocities to higher friction compared to similar films deposited on silicon wafer without wrinkles. Wear was described to be based on layer-by-layer mode, finally smoothing the wear track and reducing friction. Nevertheless, this work arises questions about the influence of the substrate elasticity (or material), the thin film material and its friction properties as well as of the contact pressure on the tribological conditions, which will be addressed in this work.

In our bio-inspired material design, we will proof the biomimetic concept of adaptability of soft materials with hard surfaces to the counterpart by fracture resulting in “pads” for ploughing (scratching) loading conditions and will investigate low- and high-cycle tribological sliding fatigue modes in dependency of the loading condition, substrate, film, film thickness, and load. Therefore, we have chosen model systems based on very thin hard films (20 - 100 nm) of titanium nitride (TiN) and a-C:H on ultra-soft, highly viscoelastic thermoplastic polyurethane (PU) in comparison to much less elastic, harder polycarbonate (PC) substrates. Because these materials were deposited differently to our previous results, their wrinkling topography and formation mechanisms are initially explained. For studying low as well as high cycle tribological fatigue, a wide range of loads (contact pressures) and differently sharp indenters (diamond tips, sapphire balls) were applied.

## 2. Experimental

### 2.1 Film deposition

TiN and a-C:H hard films were deposited at room temperature in an industrially-scaled vacuum coater (manufacturer: Pfeiffer Vacuum, Asslar, Germany) by means of pulsed laser deposition (PLD) and direct deposition from linear ion sources (plasma-activated chemical vapor deposition). PC (Senova SenolexTM, supplied by Senova, Uttendorf, Austria) and PU (Chronothane AR/LT, supplied by AdvanSource Biomaterials (Wilmington, MA, US)), both in 1 mm thickness and smoothest available surface roughness, were chosen as substrate materials with following mechanical properties: PC possess an initial elastic modulus (‪ = 0) in tension of 2330 MPa, which is valid up to 0.3% strain, where the Hook's elastic range ends. Viscoelasticity changes to viscoplastic behavior at 6% yield strain (63 MPa yield stress), followed by strain hardening (0.08 MPa/%) up to 120% strain (72 MPa stress) at failure. The applied PU is a thermoplastic polycarbonate urethane grade (thermoplastic polycarbonate blocks in polyurethane elastomer) with a highly (visco-)elastic behavior. The stress-strain curve in tension is described by the following values (according to ASTM D638): 4.5 MPa stress at 50% strain, 6.4 MPa at 100%, 11.4 MPa at 200%, 19.3 MPa at 300%, and rupture at ∼800% elongation. For some investigations, silicon wafers with (100) orientation were coated too.

Before mounting the substrates inside the vacuum chamber, ultrasonic cleaning occurred in isopropanol. Dust contamination during cleaning and mounting was limited by using grey room conditions (laminar flow). Surface activation and adhesion improving pretreatment after pumping down to start vacuum (2x10^-3^ Pa) was performed in O_2_ plasma from a linear anode layer ion source (ALS 340, Veeco Process Equipment Inc., Fort Collins, CO, US) [38]. Multi-beam PLD of TiN films with 4 overlapping vapor cones from 4 Nd:YAG lasers (1064 nm wavelength, 50 Hz pulsed, 600 mJ pulse energy, 10 ns pulse duration [36]) was conducted from pure titanium targets (ASTM Grade 2, supplied by Euro-Titan, Solingen, Germany) in N_2_-Ar gas mixture. a-C:H deposition occurred from the ALS 340 with acetylene as precursor gas [37]. During the whole film process, the substrates were continuously rotated. After the deposition and demounting in laminar flow, the samples were kept in exsiccator (< 10% relative humidity, 20°C) until characterization.

Thickness measurements on silicon wafers with masked steps by stylus profilometry (Veeco Dektak 150) revealed for the stoichiometric TiN films 100.2 nm thickness (50.2 nm thickness for samples for TEM imaging), for a-C:H films 20.3 and 100.7 nm thickness, respectively. TiN films are quite stoichiometric based on golden color and ∼50 at.% N obtained in X-ray spectroscopy measurements. Investigated a-C:H films contain a sp^2^/sp^3^ bonding ratio of ∼0.4, measured by ultraviolet Raman measurements [39], and ∼20 at.% hydrogen, measured by elastic recoil detection analysis [39]. The mechanical properties (hardness H and elastic modulus E) were measured in earlier works on silicon substrates for >1 µm film thickness and found to be H = 19 GPa, E= 170 GPa for a-C:H [39] and H = 25 GPa, E= 205 GPa for TiN [40]. Data on the compliance of these films deposited on polymers is given in [32].

### 2.2 Film characterization

Surface inspection was performed by atomic force microscopy (AFM, DI Dimension 3100 and Asylum Research MFP 3D) in tapping mode with Olympus AC 160 TS silicon tips (tip radius < 15 nm). High resolution transmission electron microscopy (HR-TEM) of the thin films was performed on a TECNAI F20-TWIN device with 200 kV acceleration voltage following thin foil preparation by microtom cutting.

Micron-scale scratch tests were performed on a NanoScratchTester (NST, CSM-Instruments, Peseux, CH) with a very sharp, cone-shaped diamond indenter (tip radius: 3 µm, cone angle: 90°). The accuracy of scratch testing on polymers was improved by 3-pass testing based on the protocol devolved by [41]: The first pass was used as topography scan to measure the initial surface conditions (topography load 0.03 mN), the second scan for scratching with 0.15 mN s^-1^ loading rate up to 10 mN maximum load at 16.6 µm s^-1^ indenter speed, and the third pass as topography scan measuring the residual plastic deformation (topography load: 0.03 mN). Pass 1 was automatically subtracted from pass 2 and 3 in order to eliminate roughness influence to improve the determination of critical loads L_c1_ and L_c2_.

Tribological sliding testing occured by linear reciprocating sliding of sapphire (Al_2_0_3_) balls (2.5 mm radius, E = 345 GPa, ν = 0.275) radius at higher loads (20 and 50 mN) and 3 mm s^-1^ speed. The contact pressures for these test conditions were estimated to be 16 to 21.5 MPa for PC and 4.0 to 5.5 MPa for PU by applying the Hertzian assumption [42] for substrates. Thin deposited films were not considered in this rough estimation. Wear investigations occurred either after a change of the friction coefficient or at the end of tribological and scratch testing by light microscopy (LIMI, Zeiss Axio) and scanning electron microscopy (SEM, Zeiss).

## 3. Results and discussion

### 3.1 Surface topography, wetting and film adhesion

**Figure 1:**
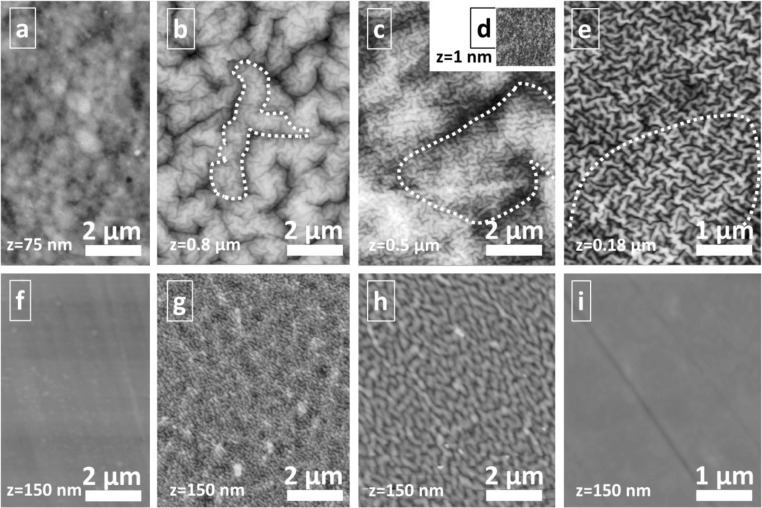
AFM images of surface topographies of uncoated substrates (a: PU, f: PC) and for wrinkle structures formed by coating with (b, g) 100 nm thin TiN films, (c, d, h) 100 nm a-C:H, and (e, i) 20 nm a-C:H. Hierarchical superstructures are marked with dotted lines.

The surface topographies (Fig. 1) for both TiN and a-C:H films on PU and PC polymers are formed by intrinsic stress induced self-assembling by nano-wrinkling, as described briefly in the introduction section and detailed in former works [30–32]. In contrast, films on stiff silicon wafers are flat and fully reproduce the substrate surface (see AFM images in Fig. 1d). The topographical features on the polymers are vermicular-like wrinkles with narrow distribution of sizes, which cover the whole surface with random orientation and high density. As visible for ultra-soft PU by comparison of the AFM images for 20 and 100 nm a-C:H films (Fig. 1c, d and e), the nano-wrinkled surface topography is formed hierarchically: Wrinkling occurs step-wise at distinct film thicknesses by mechanical instability in order to release intrinsic compressive growth stresses [31]. Consequently, the small wrinkle topography found for 20 nm films is also present on the thicker 100 nm films. Nevertheless, stiffening of the surface and reasonably higher deformation resistance for intrinsic stress relaxation leads to the introduction of additional wrinkle structures of much larger wavelength. As shown in Fig. 1e, slight indications (height differences) for the introduction of such superstructures are even present in the 20 nm thin a-C:H film. Similar feature of two different sizes of visible wrinkles is also evident for the 100 nm TiN film on PU.

For harder and stiffer PC, no formation of larger hierarchical structures is visible. Wrinkling starts above 20 nm film thickness and formed wrinkle structures at 100 nm film thickness are quite comparable to that found for 20 nm on PU (compare Fig. 1c, e and 1h, i). As described in former works [Xxx], introduction of additional larger wrinkle structures requires very soft (“ultra-soft”), easily deformable substrate materials like PU: The about one order of magnitude lower elasticity of the PC substrate shifts the introduction of larger wrinkle structures to higher film thickness than 100 nm.

**Figure 2:**
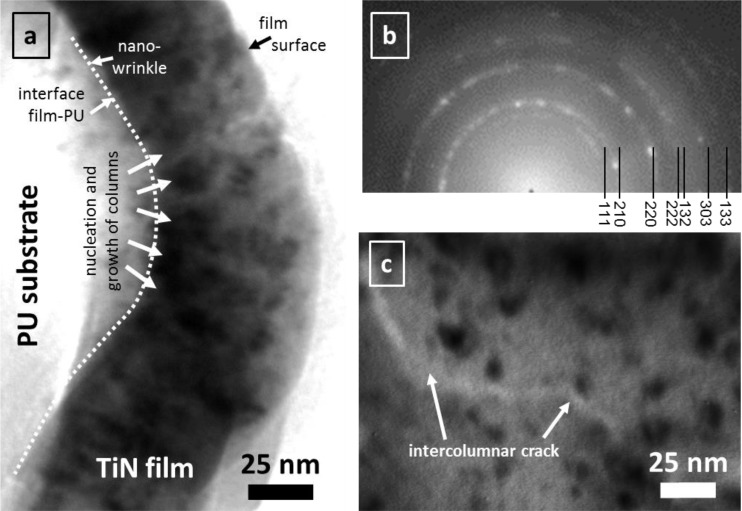
HR-TEM images of 50 nm thin TiN thin films on PU: (a) Cross-section image with arrows indicating the intercolumnar areas. (b) Small angle electron diffraction pattern. (c) Top-view image with indicated intercolumnar crack.

Generally, all nano-wrinkled surfaces are coated with dense films and film porosity is very low: Fig. 2a shows the microstructure of a 50 nm thin TiN film on PU with nanocrystalline structure (Fig. 2b). Cracks are missing in the strongly bend wrinkle, which is obviously formed by widening of the cone shaped crystallites during growth. Intercolumnar boundaries are the mechanically weak paths for film fracture (with possible nano-porosity), being aligned nearly perpendicular to the substrate surface (see arrows in Fig. 2a). Fracture along these weak paths is shown in Fig. 2c for a 50 nm TiN film, which was fragmented during too much straining in thin foil preparation by Microtom cutting. In contrast, a-C:H films are fully amorphous without any visible crystallite structures. Nevertheless, wrinkle formation is based on similar stress relaxation mechanisms for a-C:H too.

Wrinkling effects are visible by comparing the roughness values in Table 1 too: While on Si all films are perfectly smooth, roughening by a factor of 9 is found for 16x16 µm^2^ large investigated areas of 100 nm thick TiN and a-C:H films on PU, while only an increase by factor 3.5 in the roughness arises by wrinkling on PC substrates. Other effects of film growth, like formation of large grains or columns, can be excluded due to a generally higher trend for formation of such features on perfectly smooth silicon at the applied low temperatures [Lackner habil]. On the lower scale (AFM scan size 2x2 µm^2^) the hierarchical formation of wrinkles is proved by much lower RMS roughness (e.g. found for 100 nm TiN on PU).

**Table 1:** Root-mean-square (RMS) roughness of thin films deposited on PC, PU, and silicon substrates, measured by AFM and given in dependency of the scanned area size (statistics of 3 measurements).

RMS roughness [nm]					

Substrate	PC		PU		Si
AFM scan size [µm^2^]	16x16	2x2	16x16	2x2	2x2
Uncoated	6.2 ± 0.5	0.6 ± 0.1	8.2 ± 0.4	1.4 ± 0.1	0.1 ± 0.1
100.2 nm TiN	21.9 ± 1.3	18.2 ± 0.7	72.8 ± 7.9	47.8 ± 0.1	0.3 ± 0.1
20.3 nm a-C:H	12.7 ± 0.8		24.4 ± 0.8		0.5 ± 0.1
100.7 nm a-C:H	20.2 ± 1.1		73.6 ± 6.2		0.1 ± 0.1

### 3.2 Scratching and ploughing of hard films on polymers with low loads and sharp indenters

**Figure 3:**
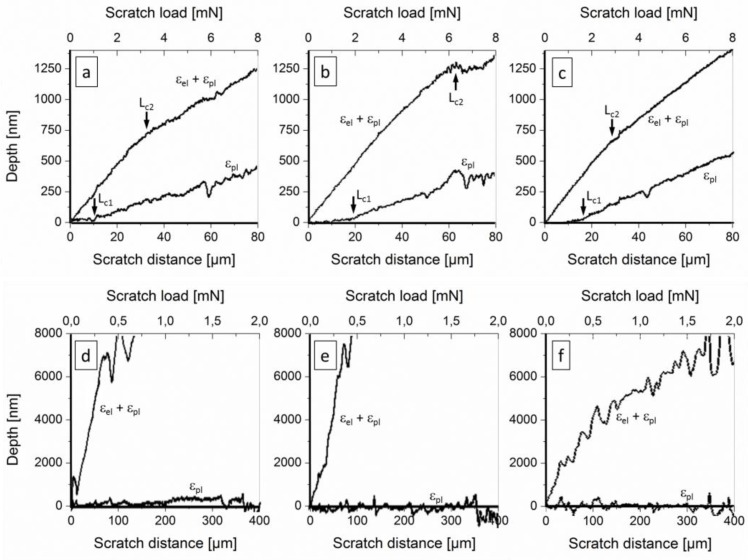
Dependency of the residual plastic deformation (ɛ_pl_) after scratching as well as the total elastic and plastic deformation during scratching (ɛ_el_ + ɛ_pl_) on distance and load during scratching with progressive loads. Critical loads (L_c1_ and L_c2_, average values of 5 scratches) are roughly indicated (for their definitions see text). Film types: (a, d) 100 nm TiN, (b, e) 20 nm a-C:H, (c, f) 100 nm a-C:H. Substrate types: (a-c) PC, (d-f) PU.

Scratch testing with progressive loads was applied in order to obtain information of forces, being necessary for cohesive and adhesive film failure on PC and PU substrates under strong ploughing conditions. During scratching, the sharp ball-shaped tip of the indenter is ploughing the surface and forming a groove due to elastic and plastic deformation, if the load bearing capacity is exceeded. The applied measurement test procedures, which include a pre- and post-scan to subtract surface roughness, enable the calculation of the residual plastic deformation (ɛ_pl_) after scratching as well as the total elastic and plastic deformation during scratching (ɛ_el_ + ɛ_pl_), as shown in Fig. 3.

#### 3.2.1 Scratching of coated PC

**Figure 4:**
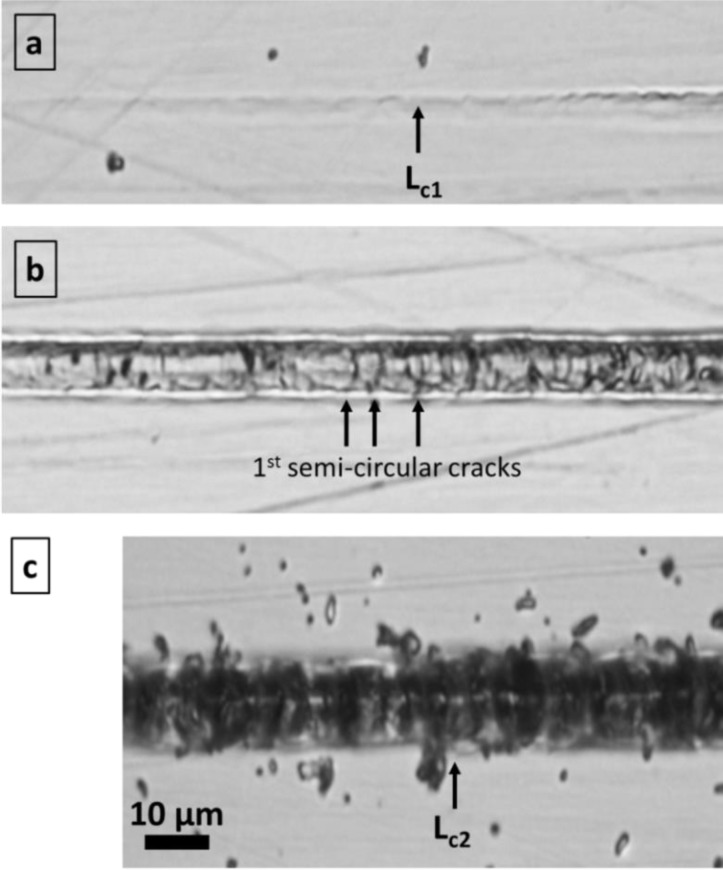
Typical failure modes of hard coatings on polymer substrates in scratch testing with progressive loads in the mN range and very sharp indenter (3 µm tip radius), shown for a 100 nm a-C:H film on PC. (a) Start of plastic deformation of the PC substrate and cohesive film fracture in the scratch track close to the edge (L_c1_). (b) Start of cohesive film fracture by transverse semi-circular cracks in the scratch track. (c) Start of adhesive fracture (chipping at edge of scratch track) between film and substrate (L_c2_).

For PC, we found independently of the type of the applied coating the well-known material response in scratching for such film-substrate material systems (Fig. 3a–c and Fig. 4): After exceeding the load-bearing capacity of the surface with (visco-)elastic, reversible contact between the diamond tip and the coated PC, plastic substrate deformation lead to permanent deformation of the compound (Fig. 4a): This is correlated with a break in the ɛ_pl_ curves (Fig. 3a–c) and first fracture of the film at the track edge parallel to the scratch direction (Fig. 4a). This event is also referred to L_c1_ in this work due to the difficulty of determination of the onset of film fracture in these material systems by the very low stored elastic energy, being released at these fracture events. Physically, this assumption for L_c1_ definition is justified by the loss of load bearing capacity of the surface. The higher L_c1_ for 100 nm thin a-C:H stands for higher ultimate strength and toughness compared to TiN, most probably due to the amorphous a-C:H vs. the nano-crystalline, nano-columnar TiN structure (grain size < 10 nm) (see above). Additionally, the elastic modulus for a-C:H is much lower (see chapter 2.2): Based on scratch test modelling by [43] for coated steels, more flexible a-C:H films can decrease the tensile stress levels below the indenter by 70% compared to TiN. Angular cracks outside the scratch track edge in the strongly bent surface zone, which are typical for higher loads and less sharp indenters [25, 44–48], are missing in the applied test protocol with mN loads. The onset of transverse semi-circular cracking in the track (Fig. 4b), generally being the next step in scratch failure, was microscopically found at lower loads for 100 nm TiN films (2.2 mN) than for 100 nm a-C:H films (2.7 mN), but isn't visible in the graphs in Fig. 3. Adhesive fracture of films on the substrate surface occurs on the scratch track edges by delamination (Fig. 4c). Starting at critical loads L_c2_ (Fig. 3), film peeling (delamination) is continuously spreading at rising loads. The initial slope of the depth-load curve (ɛ_el_ + ɛ_pl_) suddenly decreases after film delamination at L_c2_ = 2.3-2.5 mN loads for 100 nm TiN and 20 nm a-C:H, but ∼6 mN load for 100 nm a-C:H film. The position of peeling during scratching can be linked with the peak plastic deformation of the substrate at an angle of about 45° from the plane of symmetry in the plane of the film [49].

#### 3.2.2 Scratching of coated PU

While the explained behavior during scratching of films on PC is not surprisingly, the higher elasticity of PU drastically changes the occurring material deformation mechanisms towards the biomimetic materials concept: Pressing the sharp diamond indenter on the coated PU, extensive elastic substrate deformation occurs independently of the deposited film, as visible in the curves for total deformation (ɛ_el_ + ɛ_pl_) in Fig. 3d–f. Consequently, both the ball-shaped tip as well as the indenter cone surface penetrates the PU, whereby the penetration depth is about 80 times higher than for PC.

**Figure 5:**
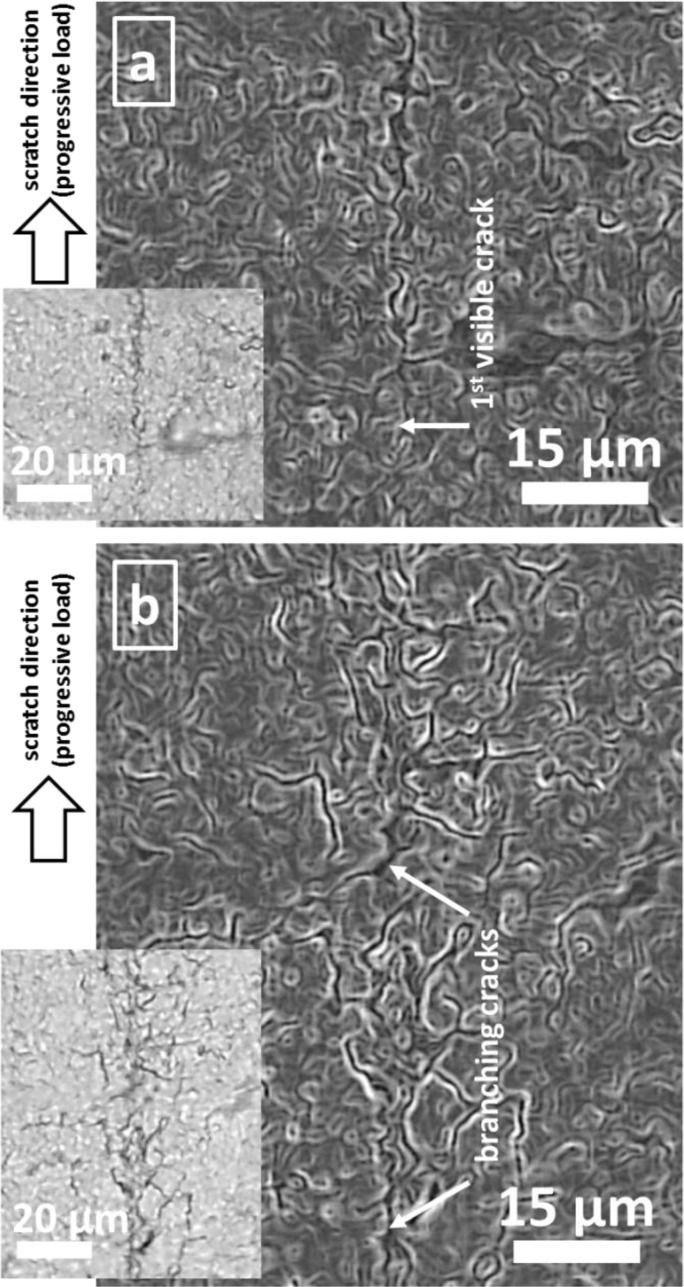
Images of scratches in 100 nm a-C:H coated PU at (a) ∼0.5 mN and (b) ∼2 mN. Scratching was performed bottom up with a diamond indenter with 1 µm tip radius. Image (a) was taken in the region of first visible fracture inside the scratch track and (b) at a position, where the crack network branched in the highly visco-elastically deformed region around the scratch without any film delamination. Overview inserts, taken by light microscopy, show clearly the crack path by the dark contrast, while charging of edges in SEM improved the visibility of the zigzag crack path following valleys of the wrinkled structure.

Under such conditions, cohesive film fracture starts under very low critical loads. Very low dissipated energies prevent experimental measurement of critical loads even by the NST device. On the contrary, the high (visco-)elasticity of PU prevents the visibility of a pronounced, permanently plastically deformed scratch track after scratching (Fig. 5). Instead of a broad track with nearly parallel edges (as shown for coated PC above), a thin zigzag line evidences the performed scratching on PU at low normal loads (Fig. 5a). Higher loads result in a branched network of zigzag lines (Fig. 5b). Hence, after the scratch contact the reversible (visco-)elastic deformation of the PU substrate closes the crack edges rather completely. The plastic deformation depth of the scratch track ɛ_pl_ (Fig. 3d–f), measured immediately after scratching, is <0.25 µm at 1 mN loads. Comparing this values to >12 µm total elastoplastic deformation (ɛ_el_ + ɛ_pl_) during scratching alludes to the very high PU volume, which is being (visco-)elastically squeezed to an uparched fold around the indenter. Nevertheless, the 100 nm a-C:H film in Fig. 5b shows no evidences for film delamination around the zigzag cracks even at higher loads, while such slight tendency to delamination was found for TiN films. This seems to have similar reasons as the lower L_c1_ value of this film on PC substrates, especially the lower toughness and the higher elastic modulus of TiN as discussed above.

Explaining the deformation mechanism for coated PU under these loading conditions in Fig. 5 requires the comparison of the micron-scale topographical relief, shown in Fig. 1c, d: The zigzag scratch fracture at low loads (Fig. 5a) follows the hierarchically largest wrinkle size on the surface, whereby the crack path follows the local mechanically weakest pathways in the wrinkle valleys. Likewise, the branched path of cracks at higher loads (Fig. 5b) runs along valleys too. Mechanically, the wrinkle valleys possess highest stress concentration in tensile straining of the surface, being present in all areas being bent and drawn during scratching. Even in less distinct hierarchical superstructures, found for 20 nm a-C:H, similar mechanisms crack paths are found.

Similar fracture mode of thin hard films on PU substrate materials were found by the authors in in-situ SEM investigations of linear tensile straining [32]: Briefly explained, uniaxial tensile load breaks TiN films on PU to segments. Cracks are generally located in the wrinkle valleys. Tensile strain is concentrated in the PU substrate below these cracks, while the substrate surface below the segments is rather unstressed. Reason therefore is the cohesion strength. Rising the uniaxial tensile stresses consequently increases stress and strain in PU bulk. Strain hardening in the elongated PU below the crack spread surface strains to larger PU volume and, thus, also to the PU surface beneath the film segments. Partition of segments is then caused by cohesive film fracture after locally exceeding film cohesion strength. The formed zigzag crack path again runs along wrinkle valleys of the largest hierarchical superstructure. Reasonably, the mechanism of zigzag fracture on a nano-wrinkled, coated soft polymer is similar for both uniaxial straining and scratch testing. The mechanism is elastically reversible and cracks close almost entirely, if no film fragments are clamped between the crack edges. Finally, the initial surface topography is restored, similar to a self-healing process. Even cyclic loading is possible [32], being important for the cyclic tribological loading of such materials, as discussed below. Finally, this fracture mode is widely biomimetically comparable to skin deformation.

### 3.3 Tribological fatigue by sliding of smooth large counterparts in dependency of contact pressure

**Figure 6:**
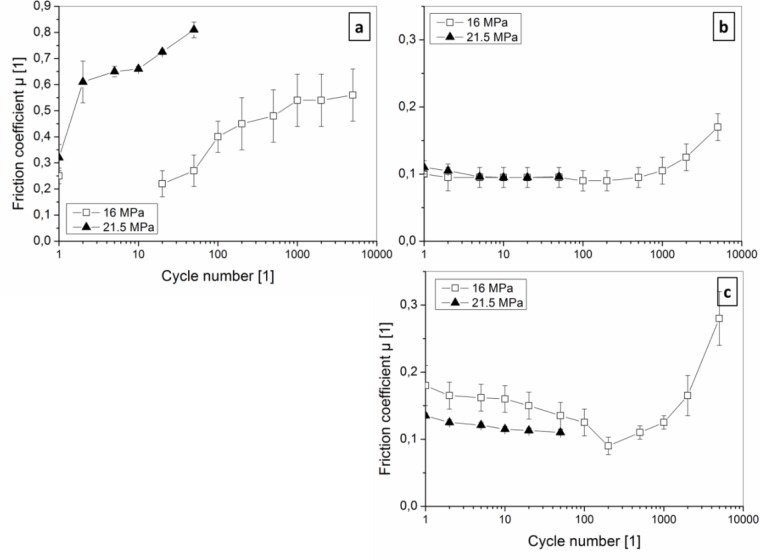
Friction coefficients in dependency of the contact pressure and the contact cycles in linear sliding of an Al_2_O_3_ ball with 2.5 mm radius for (a) 100 nm thick TiN, (b) 100 nm a-C:H and (c) 20 nm a-C:H films on PC substrates.

Based on the knowledge of compound failure mechanisms under ploughing conditions in single-pass scratch testing, tribological sliding experiments with high cycle numbers were performed with smooth, large Al_2_O_3_ ball counterparts (2.5 mm radius) and higher loads. Such conditions decrease the contact pressure down to elastically sustainable stresses for both PC and PU. Nevertheless, the occurring surface strains of >2% and >25%, respectively, are mechanically critical for the films on the polymer. Finally, maximum stresses are shifted deeper inside the polymer bulk under these contact conditions: Under assumption of the Hertzian theory, they are located in about 50% of the indenter radius below the indenter. Friction curves, given in Fig. 6 for coated PC and 7 for coated PU, reflect the reduced tendency to adhesive film failure and lower wear, enabling high cycle numbers at low friction coefficients in linear sliding tests, especially for a-C:H films.

While scratching conditions with deep penetration of the indenter lead to general high friction coefficients (0.2-0.45 at 2 mN to 0.45-0.55 at 10 mN with the higher values for TiN), sliding conditions lower initial friction at the first pass (0.2-0.4 for TiN and 0.1-0.2 for a-C:H) for both coated PC and PU. Although the friction coefficients are on similar level, the friction and wear mechanisms are very dependent on the substrate material and implicated by the substrate elasticity, illustrating biomimetic material design possibilities too.

#### 3.3.1 Sliding on coated PC

For PC substrate with lower elasticity, we found tribological effects similar to sliding on coated rigid substrates: Briefly explained, after the run-in period, either immediately increasing friction for TiN films (Fig. 6a) or decreasing friction for a-C:H films (Fig. 6b, c) is evident.

**Figure 7:**
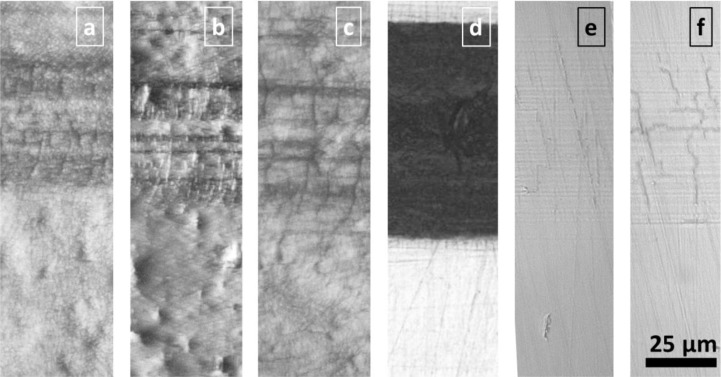
Light microscopy images of the wear track after 2000 sliding cycles of an Al_2_O_3_ ball with 5 mm diameter at 20 mN load (4 MPa contact pressure for PU and 16 MPa for PC) on coated (a-c) PU and (d-f) PC substrates. Film types: (a, d) 100 nm thin TiN, (b, e) 20 nm a-C:H, and (c, f) 100 nm a-C:H.

The slight decrease of friction and standard deviation of friction for a-C:H films is due to surface smoothing (ironing) by the sliding counterpart. The lower the load (contact pressure), the longer the period of continuous atom-scale material transfer from a-C:H film roughness tips to valleys is. The obtained friction coefficients of 0.1 to 0.2 are similar to that on rigid and smooth Si wafers. After >1000 contact cycles friction increases for a-C:H, which is based on similar mechanisms, being decisive for rising friction on TiN at much lower cycle numbers: Films are partly delaminating, the polymer surface is bared and rough wear track surfaces are formed. Such roughness is indicator for material failure below the interface in the PC. The tribological fatigue is load dependent, occurring at 20 mN load (16 MPa contact pressure, Fig. 7d) after about 100 contacts, but for 50 mN (21.5 MPa contact pressure) immediately after the first contact cycle. After film delamination – occurring most probably at or below the film-PC interface – friction coefficients are similar to the contact of Al_2_O_3_ to uncoated PC (0.3 – 0.4). As mentioned, friction for a-C:H films rise slowly, being indicator for mild fatigue: The wear track after 2000 contact cycles (Fig. 7e, f) shows some cohesive film cracks due to the cyclic (visco-)elastic PC substrate deformation, but any large adhesive fracture around the cracks is missing both for 20 and 100 nm film thickness. More pronounced fracture of the 100 nm film indicates its lower flexibility too. The initially mild wear is based on a continuous layer-by-layer removal, supported by continuously rising scratching (ploughing) by loosened particles. This is indicated by parallel sliding lines, which finally bare the PC substrate in the whole contact region. Main impact on the different behavior of TiN and a-C:H films emanates from both the material structure (nanocrystalline vs. amorphous growth) and the adhesion forces to the Al_2_O_3_ counterpart: If frictional forces are kept low (e.g. at for a-C:H and the lower normal load conditions for TiN), the shear load in the film, at the interface and below the polymer surface is low. Lower von-Mises stresses are accompanied by lower front and transverse pile-up regions during sliding, as simulated by Kral and Konvopoulos [50]. Finally, this delays fatigue mechanisms and provide high-cycle low-friction sliding.

#### 3.3.2 Sliding on coated PU

**Figure 8:**
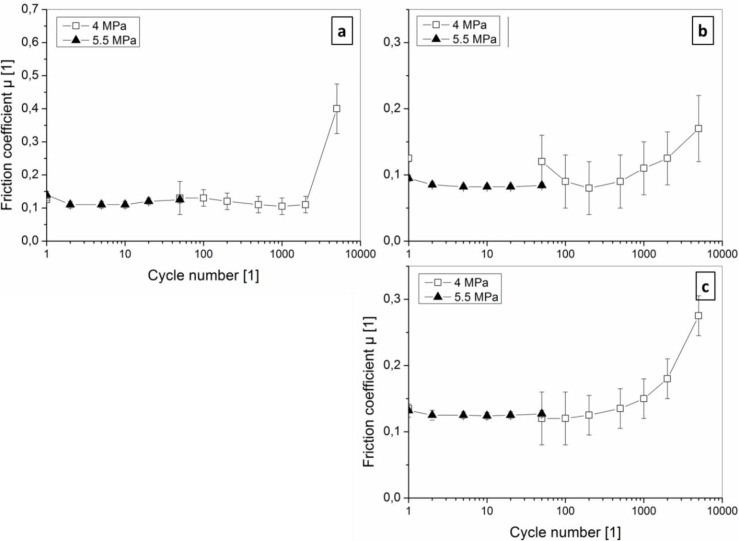
Friction coefficients in dependency of the contact pressure and the contact cycles in linear sliding of an Al_2_O_3_ ball with 2.5 mm radius for (a) 100 nm thick TiN, (b) 100 nm a-C:H and (c) 20 nm a-C:H films on PU substrates.

The tribological behavior of coated PU substrates is significantly different (Fig. 8): Friction coefficients are generally low (< 0.15) for both TiN and a-C:H films over the whole cycle number, but significantly influenced by the film thickness and the applied loads (contact pressure). Higher friction emerges for the 20 nm a-C:H thin film, although it is smoother than the 100 nm film and has a more homogenous nano-wrinkled topography with only slight hierarchical wrinkle superstructure. Apparently, this results from higher real contact area by less load-bearing capacity and better adaptation to the counterpart curvature, indicated by a denser network of smaller cracks in Fig. 7b (20 nm a-C:H) compared to Fig. 7c (100 nm a-C:H). As described above, the deformation of the substrate is concentrated below these zigzag cracks, which run along wrinkle “valleys” too. TiN films are segmented to smaller fragments and have a higher density of cracks compared to a-C:H. Nevertheless, the friction coefficient is rather similar. Missing delamination by shear cracking of the substrate, as found above for PC, enables for TiN coated PU substrates similar low friction behavior than found for a-C:H.

The comparison of friction coefficients from coated and bare (e.g. after 5000 contact cycles on TiN) or uncoated PU surfaces, shows the huge impact of even thin films and the basis for our bioinspired material design: Uncoated PU shows under similar test conditions in dry sliding generally high friction coefficients > 0.6: They are mainly due to contributions of both adhesion on molecular level [51, 52] and internal damping and energy loss in the viscoelastic body of the elastomer (hysteretic friction) [51, 53], while viscous and cohesion (tearing) components are assumed to be very small [51, 54]. Thin films on the PU surface lower both the adhesion component by the different material combination in the contact and the hysteretic friction by slightly improved load bearing capacity, which is proved by higher friction for thinner a-C:H films.

### 3.4 Aspects of bioinspired material development for low-friction surfaces on highly elastic materials

It's obvious, that under the applied normal loads both fracture of the films to small segments (“pads”) on especially ultra-soft PU as well as high film adhesion on the substrate surface are mandatory for the described tribological behavior. The contact between the counterpart and the surface – both under ploughing and sliding conditions described above – occurs on these pads, while any visco-elastic deformation is found to be concentrated in the PU surface beneath these cracks in between the pads. To minimize shear loading below the pads, low friction on their surface is mandatory. Low friction reduces front and transverse pile-up regions around the moving counterpart too and, hence, reduce tribological strains. Amorphous a-C:H films are good candidates for such materials, because their cohesive film strength is high and their elastic modulus low too.

The function of the pads, on which the tribological contact occurs, is similar to that of the thick stratum corneum regions: For low-load conditions without any tissue trauma, sliding on human skin generally occurs on these hard stratum corneum regions, which are separated from one-another by wrinkles. Load-dependent adaptation to the counterpart curvature occurs by elastic bending, which is concentrated for low loads and small topographical features of the counterpart in the smallest wrinkle structure around groups of corneocytes, for higher loads and curvatures in the secondary and primary lines [21]. Hierarchically formed wrinkles on coated PU, separating these pads with cohesive films, have similar function: They provide elasticity of the surface, whereby the hierarchical wrinkling structure contributes to adaptation to the counterpart surface too: As described in [33], high elastic deformability (>7%) is provided by smoothing out the smallest wrinkle structure in very thin films (like the 20 nm a-C:H film). However, this influence is hard to be experimentally accessed for tribological contacts. Higher required elasticity of the PU surface is provided by the shown zigzag fracture along hierarchical superstructures, which form in thicker films.

For stiffer, harder PC, the phenomenon is much less pronounced, if observing the fracture in the wear track shown in Fig. 7f (and e): Fracture of the highly adhesive films and formation of segmented wear tracks provide the required cyclic viscoelastic deformability during sliding. Wear on both coated PU and PC occurs mainly in layer-by-layer mode and without delamination of larger particles. This confirms for the applied test conditions high fatigue resistance of the segmented films in the wear track. Finally, we found similar effects for increasing surface deformability during tribological contact for carbon-fibre reinforced epoxy composites too.

## 4. Conclusions

In the current work, we broadened our bio-inspired research of structure formation and mechanical properties of nano-wrinkled thin hard films on polymers towards the tribological behavior under ploughing and sliding conditions: Based on the mechanical behavior of human skin under compressive and tensile loads and the results of tribological tests, we established a biomimetic material model for hard surfaces with low friction coefficients on soft, highly elastically deformable substrates. Sliding in such contacts occur on pads (cohesive parts of the film with high adhesion to the soft surface), which are divided by cracks. For thin films, deposited under high energetic conditions, these cracks run zigzag along the largest hierarchical (nano-)wrinkle structures, which are formed by relaxation of compressive intrinsic growth stresses by a compound deformation of elastic substrate and hard film during film growth. Additional elasticity in such wrinkled surface may be provided by smoothing of wrinkles under tensile loading. This mechanism is similar to deformation of human skin, consisting similarly of a hard layer (stratum corneum) on a soft bulk: Thick stratum corneum regions, which are similar to the described pads of films, on which the tribological contact to counterparts occur, are separated by wrinkles, which provide the elasticity by smoothing out and slightly stressing the epidermis below.

In conclusion, this bio-inspired material concept is on the way towards many technical applications for tribological protection of ultra-soft polymers.
